# The Benefits of Osteopathic Manipulative Treatment on a Patient With Ehlers-Danlos Syndrome

**DOI:** 10.7759/cureus.38698

**Published:** 2023-05-08

**Authors:** Daniel Khokhar, Bethany Powers, Musaab Yamani, Michael A Edwards

**Affiliations:** 1 Osteopathic Manipulative Medicine, Edward Via College of Osteopathic Medicine, Spartanburg, USA; 2 Osteopathic Manipulative Medicine, Alabama College of Osteopathic Medicine, Dothan, USA; 3 Department of Family Medicine, Hospital Corporation of America (HCA) Orange Park Hospital, Orange Park, USA; 4 Department of Family Medicine, Coastal Family Medicine, Orange Park, USA; 5 Department of Family Medicine, Baptist Health, Orange Park, USA

**Keywords:** omt, connective tissue disorder, eds, osteopathic manipulative techniques, osteopathic manipulative medicine, ehlers-danlos, ehlers-danlos syndrome

## Abstract

Ehlers-Danlos syndrome (EDS) is a disorder affecting connective tissue throughout the body. Inherited through several different genetic mutations, the EDS symptoms of hyperextensibility, hypermobility, and fragility cause significant somatic and visceral issues in those affected. Chronic somatic dysfunction, pain, and systemic involvement create lifelong comorbidities and discomfort for these patients. One in every 5,000 individuals is burdened with EDS worldwide; in the US, the range has been reported to be 1/2,500-1/5,000 people. Very few patients with EDS in the literature have been documented and treated with osteopathic manipulative treatment (OMT). The objective of this case report is to describe the response of an EDS patient to outpatient OMT over a series of three office visits. The patient has verbally consented to OMT at each encounter. A combination of soft tissue manipulation, muscle energy, Still’s technique, counterstrain, and high-velocity low-amplitude (HVLA) was performed in the head and neck, thoracic, lumbar, ribs, and lower extremity regions. During the three clinic visits of this patient, OMT was performed in the same regions by the student physician under the supervision of the attending physician. At each visit, the patient was asked to report their pain levels pre- and post-treatment and assess symptom improvement using a one to 10 pain scale, as well as any subjective symptoms they are experiencing. Following each treatment, as well as at each follow-up encounter, the patient reported marked pain and symptom improvement. The objective of this case report is to describe the benefits that one patient experienced from three clinic visits. These results showed that subjective improvement in respiratory, gastrointestinal, and musculoskeletal symptoms secondary to the longstanding history of EDS may be possible through the use of OMT.

## Introduction

Ehlers-Danlos syndrome (EDS) is a group of disorders affecting collagen composition and connective tissue. Inherited through several different genetic mutations, EDS leads to 13 different subtypes and multiple clinical features seen throughout the body. The most commonly described issues include joint hypermobility, skin elasticity, and musculoskeletal manifestations. The most common subtypes of EDS are classical, classical-like, hypermobile, cardio-valvular, and vascular creating a prevalence of one in 5,000 people worldwide [1, 2, 3, 4, 5, 6]. Treatment for EDS is often limited to supportive measures with medications targeting pain, anti-inflammatory, and cardiovascular symptoms, as well as complications [7, 8, 9, 10]. Osteopathic manipulative treatment (OMT) is a potential alternative in symptomatic relief for EDS patients; however, only a few case reports have documented OMT use in this population. This may be due to recent improvements in awareness and diagnoses, as well as possible contraindications related to hypermobility and tissue fragility. The goal of OMT is to restore physiologic motion and function, and it may be particularly beneficial in patients prone to structural restrictions from atypical connective tissues [1, 2, 3, 6, 11]. This case report aims to describe the response of an EDS patient to outpatient OMT conducted in a series of three visits. The patient provided verbal consent for OMT at each encounter. Soft tissue manipulation, muscle energy, Still's technique, counterstrain, and high-velocity low-amplitude (HVLA) were performed in the head and neck, thoracic, lumbar, ribs, and lower extremity regions during each clinical visit. The student physician performed OMT under the supervision of the attending physician. At each visit, the patient reported pain levels pre- and post-treatment using a one to 10 pain scale and assessed symptom improvement, as well as any subjective symptoms they experienced.

## Case presentation

A 36-year-old male patient presented to the family medicine practice for symptoms secondary to EDS, classic type and its associated genes (Table 1). The patient’s vitals were stable and reported a generalized pain rating of 5/10. He was diagnosed with EDS in his teens through clinical findings and genetic testing that revealed a COL5A1 mutation. He presented initially for diffuse pain secondary to EDS. He reported chronic pain in his neck, back, hips, thighs, and feet, along with difficulty breathing and constipation. The patient stated that after dealing with his symptoms practically his whole life, he was interested in osteopathic manipulation and verbally consented to OMT. The patient’s somatic dysfunctions during the first clinic visit were the following: bilateral suboccipital hypertonicity, atlantooccipital (OA) joint ERLSR (extended, rotated left, side-bent right), atlantoaxial (AA) joint RR (rotated right), bilateral hypertonicity at C3-C6, trapezius hypertonicity, bilateral hypertonicity at T2-T4, T2 ERLSL (extended, rotated left, side-bent left), left ribs three to six on the inhalation dysfunction, bilateral lumbar hypertonicity, L4 NSRRL (neutral, side-bent right, rotated left), bilateral tensor fascia lata hypertonicity, right anterior talofibular ligament counterstrain point, and Chapman’s points for both ascending and descending colons. The dysfunctions were discovered through a physical exam and direct palpation.

**Table 1:** EDS Subtypes and Associated Gene Mutations Adapted from [9]. EDS, Ehlers-Danlos syndrome

EDS Subtype	Gene	Mode of Inheritance
Classical	COL5A1, COL5A2, COL1A1	Autosomal Dominant
Hypermobile	TNXB	Autosomal Dominant
Vascular	COL3A1, COL1A1	Autosomal Dominant
Cardiac-Vascular	COL1A2	Autosomal Recessive
Kyphoscoliosis	PLOD1	Autosomal Recessive

The patient was educated on the treatment options and the following treatments were performed: suboccipital release; Still’s technique for OA and AA dysfunctions; soft tissue kneading and myofascial release for cervical, thoracic, lumbar, and tensor fascia lata hypertonicity; muscle energy for the rib, thoracic, and lumbar segmental dysfunctions; thoracic HVLA for the rib inhalation dysfunction; rotary motion release for colon Chapman’s points; and counterstrain position for the anterior talofibular counterstrain point. The patient acknowledged improvement in symptomatic discomfort and pain after each treatment. The patient stated he was able to breathe better, move with less restriction, and noted a decrease in pain from a 5/10 to a 2/10 on the pain scale. The objective improvement of somatic dysfunctions was observed through direct palpatory reassessment after each technique was performed. The patient was advised to properly hydrate, regularly stretch, perform at-home core and back strengthening exercises, utilize ice for soreness, and follow-up in one week.

The patient returned nine days later. He stated the OMT provided relief from his symptoms for 48 hours, including improvement in his gastrointestinal (GI) system, but gradually the symptoms began to reappear. During the second visit, the patient’s vitals were significant for a pain scale of 6/10, tachycardia at 106 beats per minute and hypertension at 140/80 mmHg; his remaining vitals were the same as his previous visit. He stated he eagerly waited for his follow-up appointment, attributing his increased pain, tachycardia, and hypertension to stress from waiting to have OMT again. After another history intake, the patient underwent another physical exam and the following somatic dysfunctions were identified through direct palpation: regional hypertonicity three of four bilaterally in the OA joint, AA joint, trapezius, T3-T6, L3-L5, upper abdominal quadrants, tensor fascia lata, anterior and posterior proximal lower extremities; and segmental dysfunctions at OA ERLSR, AA RR, and T3-T5 NSRR. These dysfunctions and treatment options were discussed with the patient. The dysfunctions were treated with the following: suboccipital release; soft tissue kneading and myofascial release for regional hypertonicity bilaterally in the OA joint, AA joint, trapezius, T3-T6, L3-L5, upper abdominal quadrants, tensor fascia lata, anterior and posterior proximal lower extremities; muscle energy for thoracic segmental dysfunction and lower extremity hypertonicity; thoracic HVLA for thoracic segmental dysfunction; and Still’s technique at the OA and AA joints. The patient’s self-reported pain levels improved to a 1/10 after the treatment. The patient again described a relief in restrictions and increased ease in respiration. The patient was, once again, advised to properly hydrate, regularly stretch, perform at-home core and back strengthening exercises, utilize ice for soreness, and follow-up in one week’s time.

Exactly one week passed and the patient returned for his next follow-up. The patient reported that his chronic pain symptoms, GI symptoms, and mobility discomfort were relieved for about five days. Once again, the patient presented with tachycardia (111 beats per minute), hypertension (142/88 mmHg), and a pain scale of 6/10, attributing them to the same cause. After his subjective intake, a physical exam was performed and the following somatic dysfunctions were discovered from direct palpation: hypertonicity bilaterally at the suboccipital, trapezius, thoracic, lumbar, tensor fascia lata, and right foot deltoid ligament regions; and segmental rotations at OA ERLSR, AA RR, T4-T7, and L4 NSRRL. These dysfunctions were treated with the following techniques: soft tissue kneading and myofascial release for the regional hypertonic musculature, Still’s technique at the OA and AA joints, muscle energy for the segmental thoracic and lumbar dysfunctions, thoracic HVLA for thoracic segmental dysfunction, and counterstrain for the deltoid ligament dysfunction. Improvement was noted after each technique was performed, and overall symptomatic improvement was verbalized by the patient. His pain reduced to a 1/10, and his breathing ease was once again subjectively significant. The patient was advised to follow-up after two weeks if needed and to continue the recommendations given at the prior appointments. Unfortunately, the patient did not return for follow-up again due to known reasons.

## Discussion

Because OMT documentation in the Ehlers-Danlos population is sparse, we discussed the option of OMT in depth with our patient before performing any manipulation, including possible complications and contraindications. The patient was initially assessed from head to toe for somatic dysfunctions and questioned regarding any specific regions that were more symptomatic for him. He heavily complained of feeling “stiff,” dyspnea, and constipation. Recently, patients with EDS have been presenting with increased difficulty breathing and GI dysfunction. The disruption of collagen integrity brings about systemic disease as described in the literature, requiring increased interdisciplinary approaches to treatment [1, 2, 5]. Current treatment protocols include physical therapy, occupational therapy, pain management, and symptomatic treatment. Symptomatic treatment of gastrointestinal, cardiovascular, and respiratory complaints is achieved through medications. OMT may be shown to be an important interdisciplinary approach for symptomatic relief [12, 13, 14, 15]. Following manipulations in the thoracic region and autonomic system, our patient’s symptoms seemed to improve, and the improvements lasted longer with each subsequent visit [16, 17]. Further research with clinical trials would allow for evaluations of the possible contraindications and indications. The manipulations that were used were specific to the dysfunctions found in this patient and the patient’s chronic disease manifestations related to EDS [18, 19, 20].

## Conclusions

The patient was consulted to develop an OMT protocol informed by his dysfunctions and his EDS. Subjectively, the patient experienced an improvement in the symptoms related to his condition. The difficulty with assessing OMT is often in objectively identifying benefits in all patients, in addition to EDS patients specifically. In future studies, objective measurements, specific treatments, and more long-term treatment courses should be utilized to bring about more conclusive evidence of lasting OMT benefits in this patient population. This patient’s subjective improvement shows great promise in the use of OMT for EDS patients. Previously the potential contraindication of OMT with EDS patients hindered its use of it completely. Given the pathology of the syndrome, specific contraindications should be observed, such as avoidance of cervical HVLA due to possible OA/AA subluxation. However, OMT can be useful when proper techniques are performed in the proper body regions. In conclusion, though it may not be generalizable to all patients with EDS, subjective short-term benefits were observed in this patient with the sequential use of a systematically constructed series of OMTs specific to the patient.
